# Citronellal Attenuates Oxidative Stress–Induced Mitochondrial Damage through TRPM2/NHE1 Pathway and Effectively Inhibits Endothelial Dysfunction in Type 2 Diabetes Mellitus

**DOI:** 10.3390/antiox11112241

**Published:** 2022-11-14

**Authors:** Ya-Ling Yin, Huan-Huan Wang, Zi-Chen Gui, Shan Mi, Shuang Guo, Yue Wang, Qian-Qian Wang, Rui-Zhu Yue, Lai-Biao Lin, Jia-Xin Fan, Xue Zhang, Bing-Yan Mao, Tian-Heng Liu, Guang-Rui Wan, He-Qin Zhan, Mo-Li Zhu, Lin-Hua Jiang, Peng Li

**Affiliations:** 1 Sino-UK Joint Laboratory of Brain Function and Injury and Department of Physiology and Neurobiology, Department of Physiology and Pathophysiology, School of Basic Medical Sciences, Xinxiang Medical University, Xinxiang 453003, China; 2 Henan International Joint Laboratory of Cardiovascular Remodeling and Drug Intervention, Xinxiang Key Laboratory of Vascular Remodeling Intervention and Molecular Targeted Therapy Drug Development, College of Pharmacy, Xinxiang Medical University, Xinxiang 453003, China; 3 Hepatic Surgery Center, Tongji Hospital, Tongji Medical College, Huazhong University of Science and Technology, Wuhan 430030, China; 4 Hubei Key Laboratory of Diabetes and Angiopathy, Hubei University of Science and Technology, Xianning 437100, China; 5 Sanquan College, Xinxiang Medical University, Xinxiang 453003, China; 6 School of Biomedical Sciences, Faculty of Biological Sciences, University of Leeds, Leeds LS2 9JT, UK

**Keywords:** citronellal, endothelial dysfunction, NHE1, TRPM2, T2DM

## Abstract

In type 2 diabetes mellitus (T2DM), oxidative stress induces endothelial dysfunction (ED), which is closely related to the formation of atherosclerosis. However, there are few effective drugs to prevent and cure it. Citronellal (CT) is an aromatic active substance extracted from citronella plants. Recently, CT has been shown to prevent ED, but the underlying mechanism remains unclear. The purpose of this study was to investigate whether CT ameliorated T2DM-induced ED by inhibiting the TRPM2/NHE1 signal pathway. Transient receptor potential channel M2 (TRPM2) is a Ca^2+^-permeable cation channel activated by oxidative stress, which damages endothelial cell barrier function and further leads to ED or atherosclerosis in T2DM. The Na^+^/H^+^ exchanger 1 (NHE1), a transmembrane protein, also plays an important role in ED. Whether TRPM2 and NHE1 are involved in the mechanism of CT improving ED in T2DM still needs further study. Through the evaluations of ophthalmoscope, HE and Oil red staining, vascular function, oxidative stress level, and mitochondrial membrane potential evaluation, we observed that CT not only reduced the formation of lipid deposition but also inhibited ED and suppressed oxidative stress-induced mitochondrial damage in vasculature of T2DM rats. The expressions of NHE1 and TRPM2 was up-regulated in the carotid vessels of T2DM rats; NHE1 expression was also upregulated in endothelial cells with overexpression of TRPM2, but CT reversed the up-regulation of NHE1 in vivo and in vitro. In contrast, CT had no inhibitory effect on the expression of NHE1 in TRPM2 knockout mice. Our study show that CT suppressed the expression of NHE1 and TPRM2, alleviated oxidative stress-induced mitochondrial damage, and imposed a protective effect on ED in T2DM rats.

## 1. Introduction

Endothelial dysfunction (ED) is a common feature of cardiovascular diseases, and it has received much attention in the study of the pathogenesis of cardiovascular diseases [1,2]. Basic and clinical studies have shown that different types of cardiovascular diseases are often associated with ED, that is, endothelial-dependent diastolic dysfunction, which in turn can aggravate cardiovascular diseases [3]. Although the exact mechanism of ED is still unclear, oxidative stress, mitochondrial damage, and changes in endothelial inflammatory activity have been reported to be involved in the occurrence of ED.

More and more evidence has shown that ED has played a key role in the development of vascular complications in diabetes. Hyperglycemia, hyperinsulinemia, and insulin resistance lead to impaired endothelial function, including barrier dysfunction, impaired nitric oxide (NO) activity, excessive production of reactive oxygen species (ROS), oxidative stress, and dysregulation of inflammation [4,5,6]. NADPH oxidase, NOX2 and NOX4, are the main sources of ROS in endothelial cells [7]. NOX2 generates superoxide, NOX4 generates hydrogen peroxide, and uncoupled nitric oxide synthase is involved in the conversion of NO to ROS. A decrease in nitric oxide (NO) bioavailability and an increase in ROS production are the main molecular changes related to endothelial dysfunction [8]. Therefore, inhibiting the production of ROS by NADPH oxidase is considered as a potential strategy for the treatment of ED induced by T2DM.

Mitochondria play a key role in maintaining the energy metabolism of endothelial cells. High glucose triggers an excessive production of ROS, and ROS increases the endothelial cells’ cytoplasmic Ca^2+^ transient and the Ca^2+^ concentration of mitochondria, which is closely related to the reduction of NO production and the damage of mitochondria and endothelial cells [9]. Therefore, reducing mitochondrial damage and inhibiting calcium influx are two important ways of mitigating ED.

Transient receptor potential (melastatin)2 (TRPM2) is an oxidant-sensitive, Ca^2+^-permeable, non-selective cation channel that is widely expressed in mammalian tissues, especially vascular endothelial cells [10]. In terms of endothelial function, TRPM2 is involved in the regulation of barrier function, cell migration and angiogenesis. Any impairment of these functions can lead to ED and atherosclerosis in diabetic disease. It has been reported that the TRPM2 channel plays an important role in mediating ROS-induced Ca^2+^ influx that causes decreased endothelial cell barrier function and induced endothelial cell death in mouse aortic endothelial cells [11]. High glucose induces ROS production, which promotes poly (ADR-ribose) polymerase (PARP)-dependent TRPM2 channel activation. TRPM2-mediated Ca^2+^ influx triggers the release of lysosomal Zn^2+^, which is subsequently accumulated into mitochondria. The accumulation of Zn^2+^ in mitochondria stimulates the recruitment of dynamin-related protein 1 (DRP1) and drives mitochondrial division, leading to mitochondrial damage [10]. Therefore, regulating the function of TRPM2 in endothelial cells may be a new target to aim for preventing diabetes-related vascular ED caused by oxidative stress.

Na^+^/H^+^ exchanger 1 (NHE1) is a membrane transporter that exchanges an intracellular proton for an extracellular Na^+^, which predominantly maintains the homeostasis of intracellular pH value [12]. NHE1 is regulated by many factors, such as hyperglycemia, protein kinase C activity, and insulin. Under the conditions of diabetes, NHE1 activation stimulates Na^+^ to enter endothelial cells in order to exchange H^+^; Na^+^ accumulates in the cytosol, driving Ca^2+^ to enter through the Na^+^-Ca^2+^ exchanger, and ultimately leads to cytosolic and mitochondrial Ca^2+^ overload causing endothelial cell damage by impairing mitochondrial function [13]. Therefore, abnormal regulation of homeostasis in vascular endothelial cells leads to ED. In view of this, inhibiting NHE1 can reduce ED caused by Na^+^-driven cytosolic and mitochondrial Ca^2+^ overload.

TRPM2 and NHE1 have recently become significant participants in the dysregulation of ion homeostasis in cardiovascular diseases, and these channels and/or exchangers are expressed in most cell types of blood vessels. Under the stimulation of hyperglycemia, the sustained activation of these ion-transporting mechanisms leads to an excessive influx of cations, such as Ca^2+^, Na^+^, and Zn^2+^, which induces ED. Therefore, inhibiting the expression of TRPM2 and NHE1 may become a new strategy for the treatment of ED induced by diabetes [14].

Citronellal (CT, 3pyrrine-7-dimethyl-6-octenal) is an essential oil with an aromatic scent that is extracted from the citronella plant and has a variety of biological beneficial effects, including antioxidant [15], anti-inflammatory, and lipid-lowering properties [16]. Moreover, it mitigates metabolic syndrome [17] which also helps to inhibit P-glycoprotein(P-gp), a protein which affects the bioavailability and efficacy of immunosuppressants and anticancer drugs [18]. However, pharmacological functions in the field of cardiovascular diseases have rarely been reported. This experimental study aimed at answering whether CT attenuated oxidative stress-induced mitochondrial damage through the TRPM2/NHE1 signaling pathway and effectively inhibited ED in T2DM rats.

## 2. Materials and Methods

### 2.1. Reagents

Citronellal (CT, Beijing Yuke Biotechnology Co., Ltd) was purchased form Beijing, China. streptozotocin (STZ), metformin (DMBG), acetylcholine (Ach) and sodium nitroprusside (SNP) were purchased from Signal-Aldrich, St. Louis, MO, USA. The kits for MDA (A003-1-2), NO (C009-2-1), ROS (E004-1-1), and SOD (A001-3-2) were obtained from Nanjing Jiancheng, Nanjing, China. Dihydroethidium (DHE, S0063, Beyotime), ROS assay kit (ROS, S0033S, Beyotime), and JC-1-mitochondrial membrane potential assay kit (C2003S, Beyotime) were obtained from Beyotime, shanghai, China. The anti-NOX2 antibody (ab129068, Abcam), anti-NOX4 antibody (ab154244, Abcam), anti-DRP1 antibody (ab56788, Abcam), anti-TRPM2 antibody (ab11168, Abcam) and anti-VDAC (ab154856, Abcam) were obtained from Abcam, Cambridge, MA, USA. The anti-NHE1 antibody (bs-0505R, Bioss) and anti-α-SMA antibody (bsm-52396R, Bioss) were obtained from Bioss, Beijing, China. The anti-GAPDH antibody (AB0036, Abways) was acquired from Shanghai, China.

### 2.2. Animals and Treatments

Seventy male SD rats (8–10 weeks, 180 ± 20 g) were acquired from the (Zhengzhou, China). Twenty male TRPM2 wild-type (TRPM2^+/+^), and twenty male TRPM2 knockout mice (TRPM2^−/−^) (TRPM2^+/+^, TRPM2^−/−^, 8 weeks, 25 ± 5 g) were provided by Professor Jiang Lin-Hua. The mice were of C57BL/6J background. All animals were kept in a temperature-controlled environment (21 ± 1 °C), humidity (40–60%), light/darkness for 12 h, and they were free to drink and eat fodder regularly. The animal experiment protocol was granted by the Institutional Animal Care Committee of Xinxiang Medical University.

After one week of adaptive feeding, the rats were randomly divided into six groups. The control group (*n* = 10) were fed with ordinary feed during the whole period. All the other rats were fed on a high-fat and high-sugar diet (sucrose, refined lard, and egg yolk were added on the basis of a standard full-priced mixed diet, 22.5 kj/kg in calories) for the 6th to 16th week (total 10 weeks). The other five groups were (*n* = 12 per group): T2DM group; T2DM + DMBG (200 mg/kg/day) group; T2DM + CT (50 mg/kg/day) group; T2DM + CT (100 mg/kg/day) group; T2DM + CT (150 mg/kg/day) group. Both DMBG and CT were given to rats by gavage for 15 weeks from the 2nd week. The rats of the five non-control groups were injected intraperitoneally with streptozotocin (STZ, 60 mg/kg in 0.1 M citrate buffer; pH 4.5) to induce a T2DM model at the 13th week [19]. Finally, fifty rats with blood glucose levels higher than 16.7 mmol/L were considered as T2DM model rats and were included in the follow-up experiment. 

In addition, the twenty male TRPM2 knockout mice were randomly divided into two groups (*n* = 10, per group): TRPM2^−/−^ group and TRPM2^−/−^ +CT group (150 mg/kg/day, gavage for 15 weeks). The twenty wild-type (TRPM2^+/+^) mice were randomly divided into two groups (*n* = 10, per group): TRPM2^+/+^ group and TRPM2^+/+^ +CT group (150 mg/kg/day, gavage for 15 weeks). The TRPM2^−/−^ group and The TRPM2^+/+^ group were fed with ordinary feed.

After 16 weeks, all animals were anesthetized with 0.4% pentobarbital for subsequent experiments on vascular function, pathological morphology, molecular biology.

### 2.3. Cell Culture and Treatment

Human umbilical vein endothelial cells (HUVECs) were obtained from the American Type Culture Collection Center (ATCC, Manassas, VA, USA). The cells were grown in endothelial cell medium (ECM, ScienCELL, California, CA, USA) supplemented with 10% fetal bovine serum (FBS) (ScienCELL, California, CA, USA), 100 μg/mL streptomycin, and 100 μg/mL penicillin in a 37 °C humidified incubator (Thermo Fisher Scientific, Rockford, IL, USA) with 5% CO_2_. HUVECs were divided into six groups: control group (cultured with normal medium); high concentration of glucose (30 mM, HG) group; HG + DMBG (50 mg/mL); HG + CT (5 μg/L); HG + CT (10 μg/L); HG + CT (15 μg/L). All reagents were added to the six different groups, respectively, and the cells were incubated in a CO_2_ incubator for 72 h.

Construct exogenous TRPM2 overexpression vector-package virus particles were used to infect HUVECs in order to obtain a TRPM2 overexpressing HUVECs model (TRPM2-o/e cells). Twenty-four hours prior to transfection, the HUVECs were plated into 6-or 24-well plates and flasks. Transfection was conducted when the cells reached 20-30% confluency. The TRPM2 lentiviral particles were used to infect cells at a multiplicity of infection (MOI) of 50. After 8 h of lentiviral adsorption and infection, the medium was removed immediately and replaced with a fresh medium containing 8% FBS. After transfection, the HUVECs were transferred to a serum-free medium and then cultured in the presence or absence of CT (10 μg/L) for 48 h [20]. The cell transfection experiment was divided into four groups: control group (cultured with normal medium); control+ CT group (15 μg/L); TRPM2-o/e group; and the TRPM2-o/e+ CT group (15 μg/L).

### 2.4. Fundus Photography

In order to observe the pathological changes of the optic disc, posterior retina and fundus arterioles, all animals were anesthetized by intraperitoneal injection of sodium pentobarbital (0.4%, 1 mL/100 g) and underwent fundus photography (optomed, VET2).

### 2.5. Vascular Morphology Observation

After the rats were anesthetized and killed, the carotid artery of each rat was stripped and made into a paraffin block, which were then cut into 5 μm thick samples. The vessels were stained with hematoxylin and eosin (HE) and Oil red respectively, to detect pathological structural changes and the formation of lipid deposits. 

### 2.6. Test of Endothelium-Dependent Relaxation (EDR)

Vasodilation of the aorta was detected using an in vitro micro-vessel culture monitoring system (DMT204CM, Danish Myo Technology, Hinnerup, Midtjylland, Danmark). The methods used in this research were adopted from the published techniques [21,22]. The aortas of the rats were dissected, and the knot and hoof tissue were removed and cut into 4 mm vascular rings. The aortal vascular rings were suspended in a glass organ channel filled with Krebs buffer. The operation method of Krebs buffer was as follows: 118.0 mM NaCl, 4.7 mM KCl, 2.5 mM CaCl_2_, 1.2 mM MgSO_4_, 1.2 mM KH_2_PO_4_, 25.0 mM NaHCO_3_, 11.1 mM glucose, pH 7.2–7.4, and 37 °C; the Krebs buffer was inflated with a mixture of 95% O_2_ and 5% CO_2._ Phenylephrine (1 μM) was applied to induce the contractile response of the aortal rings. After the phenylephrine excited a tension of the aortal vascular rings, ACh was given to induce endothelium-dependent relaxation, while SNP was given to induce endothelium–independent relaxation.

### 2.7. Detection of Diastolic Function of Mesenteric Artery

According to the method reported in the literature [19,23], the rats were anesthetized with 0.4% pentobarbital sodium (1 mL/100 g) and the mesenteric artery of each rat was dissected. The microvascular relaxation function was evaluated by comparing the relaxation ratio of the top and bottom diameters of the blood vessels in the images collected in the experimental groups.

### 2.8. Oxidative Stress-Level Assays

The contents of MDA, NO, ROS, and SOD in the carotid vasculature were measured according to the steps of the kit instructions. A dihydroethidium (DHE) fluorescent probe was used to detect the level of superoxide anion in blood vessels. An ROS assay kit was used to detect the level of ROS in cells.

### 2.9. Electron Microscope

The tissue block was washed with 4 °C pre-cooled normal saline, and quickly cut off the myocardial tissue block, with a volume of less than 3 cubic millimeters, and then fixed in pre-cooled glutaraldehyde fixed solution, dehydrated, embedded, chipped, sectioned, stained, observed mitochondria under electron microscope. Photos were taken.

### 2.10. Mitochondrial Membrane Potential Analysis

Mitochondrial membrane potential in the carotid arteries and HUVECs was assessed with the JC-1 mitochondrial membrane potential assay kit following the manufacturer’s protocol [24]. In short, the HUVECs were seeded in a 96-well black frame plate at the density of 10,000 cells/well. Mitochondrial membrane potential was indicated by the ratio of the aggregated JC-1 (red fluorescence) and the monomeric JC-1 (green fluorescence).

### 2.11. Immunofluorescence Analysis

Paraffin embedded blocks of carotid artery were cut into 8 microns, dewaxed, dehydrated, antigen heat repaired, PBS rinsed, and normal goat serum blocked. Then, sections were incubated with primary antibodies, including anti-TRPM2 (1:200, dilution), anti-NHE1 (1:100, dilution), anti-α-SMA (1:100, dilution) at 4 °C overnight. Then, the corresponding fluorescent secondary antibody was incubated at 37 °C for 2 h. Finally, the slices were photographed and preserved under a fluorescence microscope.

HUVECs were seeded in 96-well plates and treated with different factors for 24 h. HUVECs were incubated with anti-NHE1 (1:100, dilution) at 4 °C overnight. Then, they were incubated with a fluorescent secondary antibody. HUVECs in plates were imaged by fluorescence microscope.

### 2.12. Immunohistochemistry Analysis

Paraffin sections of carotid artery were dewaxed, dehydrated, blocked with 3% fetal bovine serum and incubated with primary antibodies: TRPM2 (1:200, dilution) and NHE1 (1:100, dilution) at 4 °C overnight, then incubated with the secondary antibody. The sections were stained with DAB and hematoxylin. The slices were photographed by microscope, which were then counted and analyzed by Image Pro Plus 6.0 software.

### 2.13. Fluorescence Determination of Ca^2+^ in Cell Suspension

The Fluo3-AM calcium ion fluorescent probe was used to detect intracellular Ca^2+^ concentration. The signal sampling rate was adjusted to 1 Hz with a 488 nm Xenon light (SUTTER Lambda DG-4). HUVECs cells were plated in the coverslip (diameter = 30 mm) coated by with 100 g/ml poly-L-lysine and cultured for 24 h, then were infected with TRPM2 expressing lentivirus. After 48 h, HUVECs were loaded with the calcium fluorescent dye Fluo3-AM (5 μM, Dojindo, Japan) for 30 min at room temperature in Krebs-HEPES buffer containing (in mM): 135 NaCl, 6 KCl, 2 CaCl_2_, 1.2 MgCl_2_, 10 D-glucose, 10 HEPES at pH 7.4. The coverslips were washed 3 times and cells were allowed to deesterify for at least 20 min in indicator-free Krebs-HEPES buffer before imaging. The coverslips were mounted in a perfusion chamber, fluorescent signals in cells were recorded by time series-scan imaging on microscope (Zeiss Axio Observer A1, 40×, 1.3 NA objectives) equipped with a Xenon light (488 nm light filter). Two thirds of cell somas were set as the region of interest (ROI) for image analysis. The cells were treated by CT for 5 min. The calcium signal was recorded by the real-time recorded values (*F*) at room temperature. The calcium signal was self-normalized by the real-time recorded values (*F*) and the mean baseline values recorded at the first 2min (*F*_0_) and expressed as *F/F*_0_. To determinate [Ca^2+^]_i_, high calcium solution (5 mM Ca^2+^) and Ca^2+^-free/EGTA solution were perfused respectively. The fluorescence was acquired as *F_max_* and *F_min_*. For the recorded fluorescence *F*, [Ca^2+^]_i_ = [(*F* − *F_min_*)/(*F_max_* − *F*)] × K_d_, (K_d_ = 400 nm) [25].

### 2.14. Western Blot

As described previously [26], tissues were homogenized on ice in a cell lysis buffer. The total protein of carotid blood vessels and the cells were extracted and the protein concentration was measured quantitatively using a BCA protein kit (AR1189, BOSTER). A quantity of 40 µg tissue protein or 10 μg cell protein were separated by 12.5% sodium dodecy1 sulfate polyacrylamide gel (SDS-PAGE) and then transferred to polyvinylidene fluoride (PVDF) membranes, which were blocked for 16 min using Ncmblot blocking buffer (P30500, New Cell & Molecular Biotech, Suzhou, China). PVDF was incubated with primary antibodies against anti-NOX2 (1:1000, dilution), anti-NOX4 (1:1000, dilution), anti-DRP1 (1:1000, dilution), anti-TRPM2 (1:300, dilution), anti-NHE1 (1:1000, dilution), and anti-GAPDH (1:10,000, dilution) and anti-VDAC (1:1000, dilution) at 4 °C overnight followed by incubation with horseradish peroxidase-conjugated secondary antibody for 1 h at room temperature. Finally, the protein bands were visualized by an ECL kit (P10100, Beyotime, Shanghai, China) on a ChemiDoc imaging system (Bio-Red, Hercules, CA, USA). The intensity (area X density) of the individual band on Western blots was measured by densitometry (model GS-700, Imaging Densitometer; Bio-Rad). GAPDH and VDAC served as a loading control.

### 2.15. Statistics

The data were displayed as mean ± SEM. Rat data were statistically analyzed with Graph Pad Prism 7.0 software. Multiple comparisons were analyzed with a one-way ANOVA followed by Tukey post hoc tests or Bonferroni post hoc analyses. *p*-value < 0.05 was recognized with statistical difference.

## 3. Results

### 3.1. CT Improved Arterial Stenosis and Lipid Deposition in T2DM Rats

**Figure 1:**
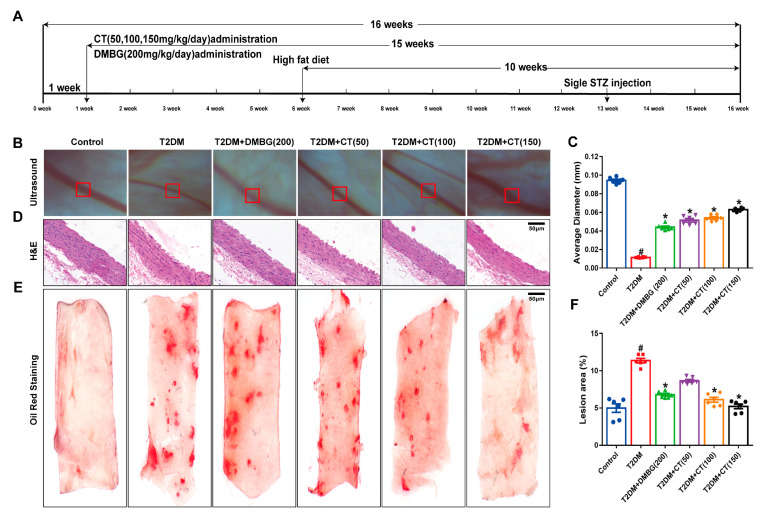
CT alleviated arterial stenosis and carotid lipid deposition in T2DM rats. (**A**) Flow chart of animal experiment. (**B**) Fundus retinal artery stenosis was examined by ophthalmoscope. (**C**) Quantitative analyses of the average diameter of retinal artery was performed. (**D**,**E**) HE staining and Oil red staining of carotid artery. (**F**) Quantitative analysis of carotid artery lipid plaque (N = 6). Data were analyzed by one-way ANOVA by Tukey post hoc tests or Bonferroni post hoc analyses. All data were expressed as mean ± SEM. ^#^ *p* < 0.05 vs. Control group, * *p* < 0.05 vs. T2DM group.

Firstly, the in vivo experiments were carried out to explore whether CT could reduce the degree of arterial stenosis and lipid deposition in T2DM rats. As shown in Figure 1A, the animals with T2DM were given CT (50, 100, and 150 mg/kg/day) by gavage for 15 weeks. Secondly, diabetic retinopathy, one of the most common complications of T2DM, is one of the major causes of vision loss all over the world [27,28]. Therefore, retinal artery stenosis was examined by ophthalmoscope to verify the effect of CT on artery stenosis. Compared with the control group, the thickness of the retinal artery wall was uneven and the average diameter of the retinal artery was significantly reduced (Figure 1B,C). Thirdly, the pathological changes of carotid atherosclerotic plaque were detected by HE staining and Oil red staining (Figure 1D,E). The area of carotid lipid deposition was remarkably increased in T2DM rats compared with the control group. Surprisingly, CT treatment could significantly reduce the stenosis of the retinal artery and the size of carotid lipid deposition in T2DM rats (Figure 1F). Compared with the T2DM group, the DMBG group could improve retinal artery stenosis, but had little effect on carotid lipid deposition (Figure 1E,F). In short, these results showed that CT could inhibit arterial stenosis and atherosclerotic plaque formation in T2DM rats.

### 3.2. CT Alleviated the Endothelial Dysfunction and Oxidative Stress in T2DM Rats

**Figure 2:**
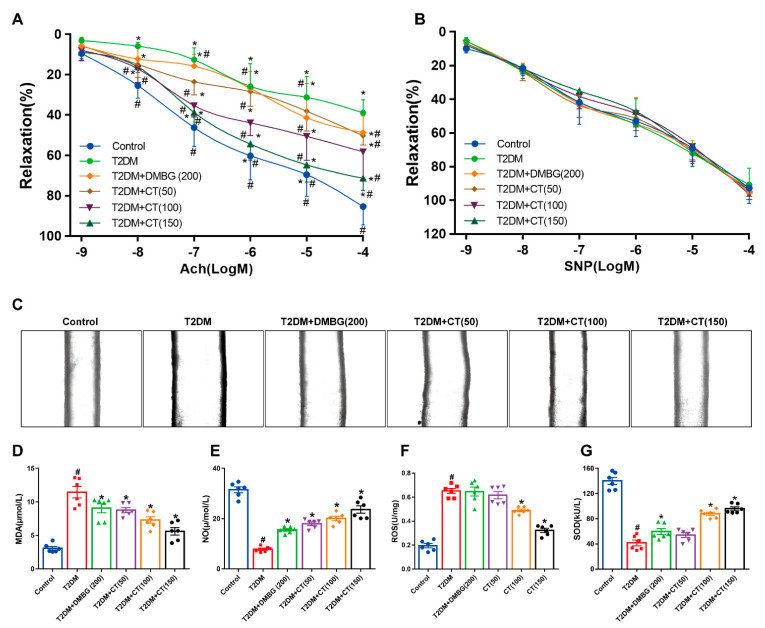
CT mitigated the dysfunction and oxidative stress of endothelial in T2DM rats. (**A**) Endothelium−dependent relaxation induced by acetylcholine (ACh) in the aortas. (**B**) Endothelium−independent relaxation induced by sodium nitroprusside (SNP) in the aortas. (**C**) Mesenteric artery diastolic function was detected by in vitro microvascular tension measurement system. (**D**−**G**) The levels of MDA, NO, ROS, and SOD in carotid arteries were examined (N = 6). Data were analyzed by one-way ANOVA by Tukey post hoc tests or Bonferroni post hoc analyses. All data were expressed as mean ± SEM. ^#^ *p* < 0.05 vs. Control group, * *p* < 0.05 vs. T2DM group.

Firstly, when endothelial lining was damaged, lipoprotein and leukocyte entered the vascular wall, which was the early key event to promote the atherosclerosis formation of T2DM [29]. Endothelial−dependent diastolic dysfunction is one of the striking features of T2DM-induced endothelial dysfunction [30], so we examined the EDR between the groups. As shown in Figure 2A, CT significantly attenuated the impaired aortic EDR response in T2DM rats in a dose dependent manner, suggesting that CT inhibited ACh−induced vascular endothelium-dependent relaxation. However, as shown in Figure 2B, the trend of CT and DMBG was consistent, indicating that CT and DMBG had no effect on SNP-induced endothelial−independent relaxation. Secondly, ED is a key factor leading to increased cardiovascular complications in T2DM [19]. The vasodilation of the mesenteric artery was detected using an in vitro micro-vessel culture monitoring system [23]. Compared with the T2DM group, CT significantly elevated the diastolic function of mesenteric artery in T2DM rats (Figure 2C). Taken together, these data confirmed that the CT could mitigate ED in T2DM rats. Thirdly, oxidative stress plays an important role in ED induced by T2DM [31,32]. It has been reported that reduced NO production in vascular tissues lead to impaired ACh−induced endothelial-dependent relaxation, which in turn affected vasoconstriction and relaxation [33]. Next, we examined whether CT antagonized oxidative stress in carotid vessels of T2DM rats. In the carotid vessels of the T2DM group, the levels of MDA and ROS were increased and the activities of NO and SOD were decreased, which were reversed by treatment of CT (Figure 2D–G).

### 3.3. CT Attenuated Oxidative Stress-Induced Mitochondrial Damage in Carotid Arteries of T2DM Rats

**Figure 3:**
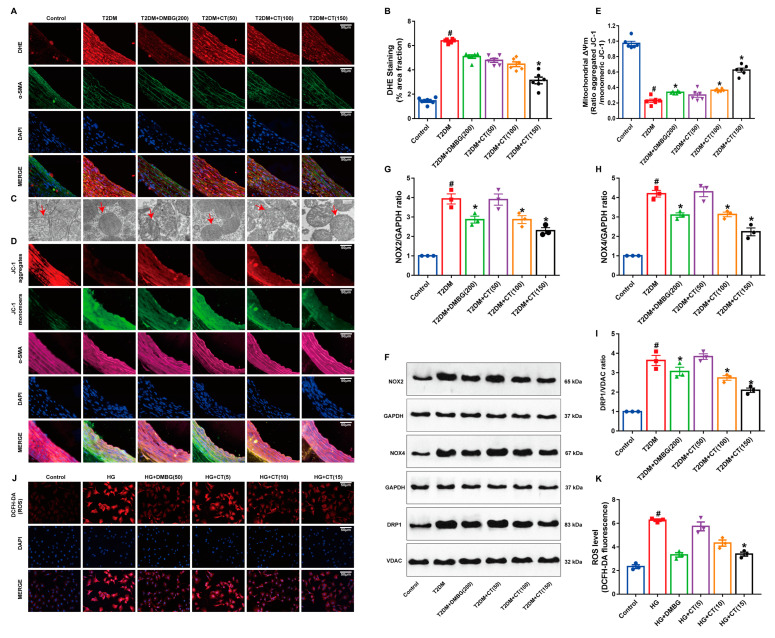
CT reduced oxidative stress-induced mitochondrial damage in carotid arteries of T2DM rats. (**A**) Detection of superoxide anion level in carotid arteries (DHE, *red*; α-SMA, *green*). (**B**) The fractional area of fluorescent assay of the arterial sections based on the measurement of the confocal microscopic images. (**C**) Ultrastructural analysis of mitochondria under electron microscope (12,000×). (**D**) Detection of mitochondrial membrane potential in carotid arteries (JC-1aggregates, *red*; JC-1 monomers, *green*; α-SMA, *purple*). (**E**) The quantitative analysis of the mitochondrial membrane potential (Δψm) (N = 6). (**F**–**I**) Western blot analysis of NOX2, NOX4, and DRP1 in carotid arteries. (**J**) HUVECs were stained with DCFH-DA (DCFH-DA, *red*). (**K**) The ROS fractional area of fluorescent assay based on the measurement of the confocal microscopic images (N = 3); Data were analyzed by one-way ANOVA by Tukey post hoc tests or Bonferroni post hoc analyses. All data were expressed as mean ± SEM. ^#^ *p* < 0.05 vs. Control group, * *p* < 0.05 vs. T2DM group.

ROS is the main factor causing oxidative damage of cells. Firstly, we determined the content of ROS in the carotid arteries. Compared with the control group, ROS was significantly increased in the T2DM group. After CT treatment, the level of ROS was significantly lower than that in the T2DM group (Figure 3A,B). Mitochondria are the main target organelles of cellular damage caused by ROS. Secondly, the structural changes of mitochondria in the carotid arteries were observed by electron microscope. In the control group, most mitochondria showed long tubular or branched structures with regular distribution. In the T2DM group, the number of mitochondria decreased significantly and were arranged in a disorderly and fragmented way. The different doses of CT could significantly improve mitochondrial injury (Figure 3C). Thirdly, the decrease in transmembrane potential in mitochondria is the earliest event in the cascade of cell apoptosis. Compared with the control group, the membrane potential of mitochondria was significantly decreased in the T2DM group, showing that CT intervention could reverse the decrease in mitochondrial membrane potential (Figure 3D,E). NOX2 and NOX4 in the NAPDH oxidase (NOX) family are the main active ROS–producing enzymes. Mitochondrial fission protein DRP1 facilitates mitochondrial fusion by adjusting its phosphorylation status at Ser616 resulting in mitochondrial dynamic distortion and even apoptosis. Fourthly, compared with the control group, the protein expression levels of NOX2, NOX4, and DRP1 were increased in the T2DM group. However, the expression levels of NOX2, NOX4, and DRP1 were significantly decreased in the CT group (Figure 3F–I). In addition, in high-glucose-cultured endothelial cells in vitro, the level of ROS was increased, but CT treatment was able to significantly reduce the production of ROS (Figure 3J,K). Therefore, CT could relieve ROS accumulation and oxidative stress-induced mitochondrial injury in the carotid arteries of T2DM rats.

### 3.4. CT Alleviated the ED by Inhibiting the TRPM2/NHE1 Pathway in Carotid Arteries of T2DM Rats

**Figure 4:**
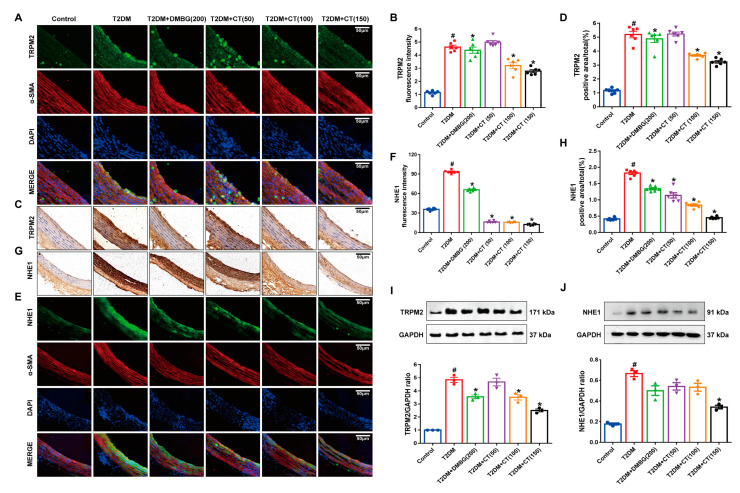
CT suppressed the protein expression of TRPM2 and NHE1 in carotid arteries of T2DM rats. (**A**) The immunofluorescence detection of TRPM2 in carotid arteries (TRPM2, *green*; α-SMA, *red*). (**B**) Quantitative analysis of fluorescence intensity of TRPM2. (**C**) The immunohistochemistry detection of TRPM2 in carotid arteries. (**D**) Quantitative analysis of positive intensity of TRPM2. (**E**) The immunofluorescence detection of NHE1 in carotid arteries (NHE1, *green*; α-SMA, *red*). (**F**) Quantitative analysis of fluorescence intensity of NHE1. (**G**) The immunohistochemistry detection of NHE1 in carotid arteries. (**H**) Quantitative analysis of positive intensity of NHE1 (N = 6). (**I**,**J**) Western blot analysis of TRPM2 and NHE1 in carotid arteries (N = 3). Data were analyzed by one-way ANOVA by Tukey post hoc tests or Bonferroni post hoc analyses. All data were expressed as mean ± SEM. ^#^ *p* < 0.05 vs. Control group, * *p* < 0.05 vs. T2DM group.

The inhibition of TRPM2 and NHE1 plays a defensive role in the protection of vascular endothelium during physiological and pathological conditions [14]. Previous studies have indicated the possibility of targeting TRPM2 as a potential therapeutic target for the formation of ROS, inflammation, and for promoting macrophage infiltration into the vascular wall [33]. NHE1 was closely related to vascular disease, especially the instability of endothelial dysfunction [34]. Therefore, we investigated the levels of TRPM2 and its downstream target protein NHE1 by immunostaining. The levels of TRPM2 (Figure 4A–D) and NHE1 (Figure 4E–H) significantly increased in the T2DM group. In contrast, CT treatment could reduce the expression levels of TRPM2 and NHE1 proteins. The DMBG group also altered the TRPM2 and NHE1 protein levels. At the same time, we used Western blotting experiments to re-verify the expression of TRPM2 and NHE1 proteins, and consistent with the previous results, CT was able to inhibit the expression of TRPM2 and NHE1 proteins (Figure 4I,J). CT may prevent T2DM-induced ED via inhibiting activation of the TRPM2/NHE1 signaling pathway.

### 3.5. CT Inhibited the Up-Regulation of NHE1 and Mitochondrial Damage in HUVECs with Overexpression of TRPM2

**Figure 5:**
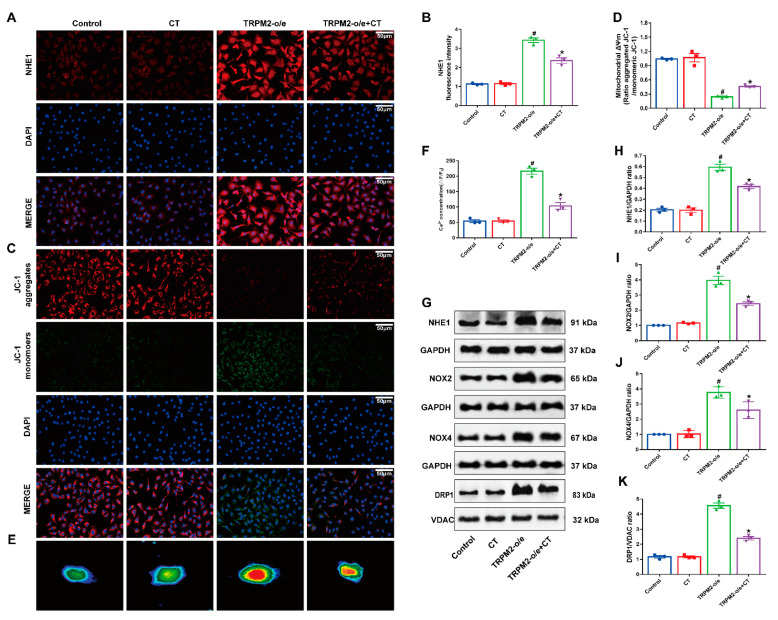
CT suppressed the expression of NHE1 protein and oxidative stress-induced mitochondrial damage in endothelial cells with overexpression of TRPM2. (**A**) The immunofluorescence detection of NHE1 in endothelial cells (NHE1, *red*). (**B**) Quantitative analysis of fluorescence intensity of NHE1. (**C**) Detection of mitochondrial membrane potential in endothelial cells (JC-1aggregates, *red*; JC-1 monomers, *green*). (**D**) The quantitative analysis of the mitochondrial membrane potential (Δψm). (**E**) Calcium concentration in endothelial cells was detected by calcium imaging technology. (**F**) Quantitative analysis of calcium fluorescence intensity in endothelial cells. (**G**–**K**) Western blot analysis of NHE1, NOX2, NOX4, and DRP1 in endothelial cells (N = 3). Data were analyzed by one-way ANOVA by Tukey post hoc tests or Bonferroni post hoc analyses. All data were expressed as mean ± SEM. ^#^ *p* < 0.05 vs. Control group, * *p* < 0.05 vs. TRPM2-o/e group.

To explore the detailed mechanism by which CT alleviated ED induced by T2DM, we used a lentiviral-mediated strategy to overexpress TRPM2 (TRPM2-o/e) in HUVECs before CT treatment. Compared with the control group, the fluorescence intensity of NHE1 protein in the TRPM2 overexpression group was significantly increased, but after 48 h of CT treatment, the fluorescence intensity of NHE1 protein was significantly decreased (Figure 5A,B). Compared with the control group, the mitochondrial membrane potential of TRPM2 overexpression group was significantly reduced, and CT treatment could reverse the reduction of membrane potential and reduce the damage of endothelial cells (Figure 5C,D). Meanwhile, we observed that the intracellular Ca^2+^ fluorescence intensity of the TRPM2 overexpression group was significantly higher than that of the control group, and CT could antagonize the increase in intracellular Ca^2+^ fluorescence intensity induced by the TRPM2 overexpression group (Figure 5E,F). Finally, although the experimental results of Western blotting showed that the expression level of NHE1, NOX2, NOX4, and DRP1 in the TRPM2 overexpression group was higher than that in the control group, CT treatment reversed these effects (Figure 5G–K). These results displayed that CT reduced mitochondrial damage by inhibiting the expression of NHE1 in endothelial cells with overexpression of TRPM2.

### 3.6. CT Inhibited the Expression of NHE1 via TRPM–Dependent 2 Signaling Pathway

**Figure 6:**
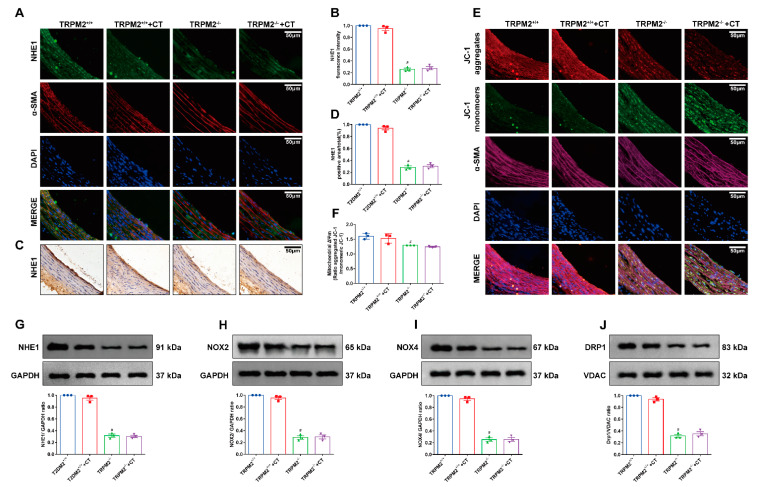
CT had no effect on the expression of NHE1 protein in TRPM2 knockout mice. (**A**) The immunofluorescence detection of NHE1 in carotid arteries (NHE1, *green*; α-SMA, *red*). (**B**) Quantitative analysis of fluorescence intensity of NHE1. (**C**) The immunohistochemistry detection of NHE1 in carotid arteries. (**D**) Quantitative analysis of positive intensity of NHE1. (**E**) Detection of mitochondrial membrane potential in carotid arteries (JC-1aggregates, red; JC-1 monomers, green; α-SMA, purple). (**F**) The quantitative analysis of the mitochondrial membrane potential (Δψm). (**G**–**J**) Western blot analysis of NHE1, NOX2, NOX4, and DRP1 in carotid arteries (N = 3). Data were analyzed by one-way ANOVA by Tukey post hoc tests or Bonferroni post hoc analyses. All data were expressed as mean ± SEM. ^#^ *p* < 0.05 vs. TRPM2^+/+^ group.

To further confirm whether CT reduced T2DM-induced ED through the TRPM2/NHE1 signaling pathway, we used an animal model of TRPM2 knockout mice. As shown in the results of immunofluorescence, immunohistochemistry, and mitochondria membrane potentials of carotid vessels, the expression of NHE1 and mitochondrial membrane potential was significantly reduced in the TRPM2^−/−^ group compared with the TRPM2^+/+^ group. Moreover, compared with TRPM2^−/−^ group, the expression of NHE1 and mitochondria membrane potentials did not change significantly in the TRPM2^−/−^+CT group (Figure 6A–F). Compared with the TRPM2^+/+^ group, NHE1, NOX2, NOX4 and DRP1 proteins were significantly decreased in the TRPM2^−/−^ group, while there was no significant difference in the expression levels of NHE1, NOX2, NOX4, and DRP1 between the TRPM2^−/−^ group and the TRPM2^−/−^+CT group by Western blotting assay (Figure 6G–J). Combined with the results in Figure 5, these findings confirmed that CT attenuated mitochondrial damage and ED via the TRPM2/NHE1 signaling pathway.

## 4. Discussion

Aromatherapy has a long history and culture in the application of folk medicine in Southeast Asia. Aromatic plant extracts have rich medicinal value and are used in medical fields such as diabetes, cardiovascular, hypertension, obesity, arthritis, cancer, and leprosy [16,35]. CT is a natural plant component with an aromatic scent. In this study, we explored the pharmacological effects of CT on T2DM-induced ED. We confirmed that: (1) CT suppressed ED in vitro and in vivo by inhibiting the TRPM2/NHE1 signaling pathway; (2) CT could reduce oxidative stress-induced mitochondrial damage.

We observed that CT could alleviate ED induced by diabetes, which was consistent with previous reports [3]. NHE1 is a key regulator of cell volume and intracellular ions under physiological and pathological conditions. NHE1 is mainly expressed in tissues of the cardiovascular system. Under the stimulation of hyperglycemia in diabetes, NHE1 is hyperactivated due to the increase in extracellular osmotic pressure, which has a harmful effect on vascular endothelial cells and promotes the formation of atherosclerotic plaques [36]. Numerous reports have demonstrated that TRPM2 is involved in ROS-induced death of hematopoietic cells, neurons, and vascular endothelial cells [11,37,38,39]. Facilitated by TRPM2, Ca^2+^ entry leads to its rapid and excessive accumulation. In turn, disturbances in intracellular calcium homeostasis lead to cell death. While apoptosis is important for maintaining homeostasis, injury repair, and organ development, in endothelial cells it also promotes pathological changes associated with inflammatory responses and vascular disease. Meanwhile, according to our previous research, NHE1 plays an important role in diabetes-induced atherosclerosis [3]. Therefore, the purpose of this study was to investigate whether CT ameliorated T2DM-induced ED by inhibiting the expression of TRPM2/NHE1 protein. The results of HE and Oil red staining showed that CT had an inhibitory effect on atherosclerosis induced by diabetes. Moreover, the results of immunofluorescence, immunohistochemistry and Western blot of rat carotid artery vessels further proved that CT played an anti-atherosclerotic role by inhibiting the expression of NHE1. Our findings agreed with drugs such as amorphous nano-selenium quantum dots [40], amiloride [41], probucol [42], and floralozone [1] which have been confirmed to prevent atherosclerosis by suppressing NHE1.

ED is an early event of vascular disease in diabetes, which is closely related to cardiovascular risk factors such as age, sex, hypertension, smoking, hyperglycemia and dyslipidemia. The hallmarks of ED are the dysregulation of endothelial nitric oxide synthase (eNOS) and the up-regulation of ROS in endothelial cells, leading to a significant decrease in the bioavailability of NO [43]. According to the reported methods [1,3,40,44], we found that CT had a protective effect on ED induced by T2DM through EDR vasodilation experiments.

Oxidative stress is a molecular disorder of reactive oxygen metabolism and plays a key role in the pathogenesis of endothelial dysfunction and atherosclerosis [45,46]. NADPH oxidase catalyzes the generation of ROS. NOX2 and NOX4 are two subtypes of NADPH oxidase, are mainly expressed in endothelial cells, and participate in the generation of ROS, superoxide, and hydrogen peroxide, all of which promote endothelial dysfunction [47]. The epidemiological investigation showed that the level of ROS in a mitochondrion was increased, the mitochondrial membrane potential was decreased, and the expression level of mitochondrial DRP1 protein was increased in patients with diabetes [48]. Our results indicated that CT down-regulated the levels of MDA, NO, ROS, and superoxide anion in carotid arteries of diabetic rats, and it down-regulated the level of ROS in endothelial cells. Moreover, CT was not only able to improve the decrease in mitochondrial membrane potential but also to inhibit the expression of NOX2, NOX4 and DRP1, thus alleviating the damage of mitochondria and endothelial cells caused by oxidative stress. These results were in accordance with previous studies [49,50,51,52].

The main mechanism of oxidative stress-induced cell damage is to disrupt intracellular ion homeostasis. TRPM2 is a calcium ion-permeable cation channel that is activated in response to oxidative stress, whereby resulting in the increase in calcium influx in endothelial cells and further causing endothelial dysfunction [53,54,55]. Meanwhile, TRPM2 may be involved in high-glucose-induced mitochondrial fission in endothelial cells [56]. The process of mitochondrial fission is strictly connected with apoptosis [57]. One of the consequences of T2DM is a high concentration of circulating glucose. It leads to the production of ROS, affecting endothelial cells and the entire circulatory system. It has been shown that inhibiting TRPM2 using its TRPM2 inhibitor or TRPM2 silencing RNA blocks mitochondrial fission in HUVECs [56]. In addition, hyperglycemia induces the activation of NHE1 on the membrane of endothelial cells, which makes the endothelial cells alkalized, disrupts the homeostasis, and triggers endothelial dysfunction [58]. Our results confirmed that CT inhibited the expression of TRPM2 and NHE1 in carotid arteries of T2DM rats. In order to verify whether CT could reduce the expression of NHE1 by inhibiting the TRPM2 signal pathway and alleviate the mitochondrial damage and ED caused by oxidative stress, we used the strategy of lentiviral transfection to induce TRPM2 overexpression in HUVECs. Our results showed that an overexpression of TRPM2 could increase NHE1 expression, decrease mitochondrial membrane potential, increase calcium concentration, and increase the expression of NOX2, NOX4, and DRP1 in endothelial cells. However, all of these effects were reversed by the treatment of CT. Meanwhile, we used TRPM2 knockout mice for the experiments. We found no significant changes in the expression of NHE1, NOX2, NOX4 and DRP1 or of mitochondrial membrane potential in TRPM2 knockout mice.

In summary, we confirmed that CT was a potential new drug that inhibited ED induced by T2DM. Its mechanism of action was related to attenuate oxidative stress-induced mitochondrial damage via suppressing the TRPM2/NHE1 signal pathway in T2DM.

## Data Availability

Data are contained within the article.
